# Influenza-associated Hospitalizations and Deaths, Costa Rica, 2009–2012

**DOI:** 10.3201/eid2005.131775

**Published:** 2014-05

**Authors:** Guiselle Guzman Saborío, Alexey Clara, Antonio Garcia, Fabio Quesada, Rakhee Palekar, Percy Minaya, Marvin Cervantes, Mariel Lopez, Jenny Lara, Jorge Jara, Natalia Blanco, Joseph Bresee, Marc-Alain Widdowson, Eduardo Azziz-Baumgartner

**Affiliations:** Caja Costarricense del Seguro Social, San Jose, Costa Rica (G. Guzman Saborío , A. Garcia, F. Quesada, M. Cervantes);; US Centers for Disease Control Central American Regional, Guatemala City, Guatemala (A. Clara);; Pan American Health Organization, Washington, DC, USA (R. Palekar);; Centers for Disease Control and Prevention, Atlanta, Georgia, USA (R. Palekar, J. Bresee, M-A Widdowson, E. Azziz-Baumgartner);; Training of Epidemiology and Public Health Intervention Network, Guatemala City (P. Minaya);; Instituto Costarricense de Investigación y Enseñanza en Nutrición y en Salud, San Jose (M. Lopez, J. Lara);; Universidad del Valle de Guatemala, Guatemala City (J. Jara, N. Blanco)

**Keywords:** influenza, hospitalization rate, mortality rate, severe acute respiratory infections, viruses, Costa Rica, tropical, Americas

## Abstract

Data needed to guide influenza vaccine policies are lacking in tropical countries. We multiplied the number of severe acute respiratory infections by the proportion testing positive for influenza. There were ≈6,699 influenza hospitalizations and 803 deaths in Costa Rica during 2009–2012, supporting continuation of a national influenza vaccine program.

During 2002–2008, 41,000–160,000 persons died from influenza-associated illnesses throughout the Americas (*1*). Although it is known that influenza is preventable by use of vaccines (*2*), documentation of the value of vaccination, including data on influenza-associated hospitalizations and deaths, is limited in middle-income tropical countries.

In 2004, Costa Rica, an upper–middle income country (*3*), recommended influenza vaccines for children 6 months–8 years of age who had pre-existing conditions and for persons ≥65 years of age (*4*) although national data to support this recommendation were limited. We used the World Health Organization International Classification of Diseases, 10th Revision (ICD-10) hospital discharge criteria (*5*), the Costa Rica National Influenza Center surveillance (*6*), and census population data (*7*) to quantify influenza-associated hospitalization and mortality rates during 2009–2012 to guide national and subregional vaccine policy.

## The Study

In 2005, Costa Rica maintained a database of persons hospitalized throughout the country’s public hospitals. Approximately 95% of all hospitalizations occur within this centralized health care system. This hospital database contains information on patients’ demographics, their first 6 ICD-10 coded diagnoses, and their survival. We used this information to determine the number of persons admitted to public hospitals during 2009–2012 and discharged with proxy diagnoses of severe acute respiratory infection (SARI) (ICD-10 code J9–18) (*8*). We used viral surveillance data to estimate the proportion of influenza-positive specimens if all had been tested by using published methods (*9*).

Influenza surveillance is conducted by Costa Rica’s National Influenza Center and the *Instituto Costarricense de Investigación y Enseñanza en Nutrición y Salud* (INCIENSA). In 2002, INCIENSA laboratory staff began testing clinical nasal and pharyngeal samples for influenza by using indirect immunofluorescence. Starting in 2007, Costa Rica systematically identified 5 SARI case-patients (defined as patients with fever and cough or sore throat with respiratory difficulties requiring hospitalization) per sentinel site per week (*6*) to supplement specimens obtained through routine clinical practice. Influenza surveillance was fully operational by the 2009 pandemic year (Technical Appendix). INCIENSA primarily tested respiratory samples through indirect immunofluorescence and subtyped positive specimens through real-time reverse transcription PCR.

We stratified the percent of influenza-positive samples among SARI case-patients into groups of patients who were <5, 5–59, and ≥60 years of age to broaden the implications of our findings for tropical countries. For each age group, we multiplied the monthly number of SARI case-patients by the proportion of influenza-positive samples from SARI case-patients (Figure) and their 95% CI (*9*).

**Figure:**
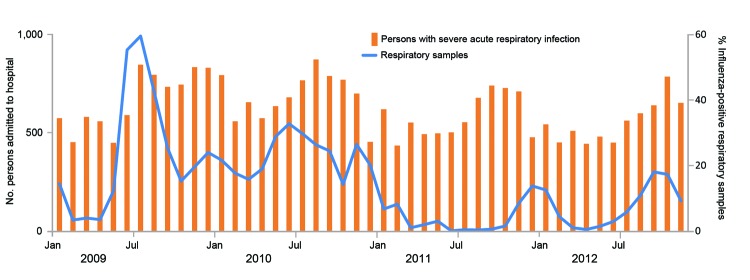
Hospitalizations for severe acute respiratory infection and proportion of samples testing positive for influenza in Costa Rica during 2009–2012. National Influenza Centre and influenza sentinel surveillance sites in the provinces of San José, Costa Rica.

We assumed that all persons living in Costa Rica were at risk for development of SARI and being admitted to a public hospital. We divided the estimated annual number of influenza-associated hospitalizations and deaths by the census projections for each year and age group (*7*). We stratified our influenza analyses into pandemic (2009–2010) and seasonal influenza years (2011–2012) because we anticipated that case ascertainment, health utilization, hospital admissions, respiratory specimen collection, and rate estimates would be different during these periods. For example, during 2009, to help the World Health Organization better characterize a novel virus, surveillance staff supplemented systematic with snowball sampling of the contacts of persons in whom diagnoses of influenza A (H1N1)pdm09 virus were laboratory confirmed (*10*). The Institutional Committee of Bioethics in Research approved this program evaluation.

During January 2009–December 2012, we identified 30,357 persons hospitalized for treatment of SARI throughout Costa Rica, of whom ≈1/3 of case-patients belonged to each age group (29% <5 years of age, 32% 5–59 years of age , and 38% ≥60 years of age). Deaths associated with SARI occurred primarily among persons ≥60 years of age. Although 1% of children <5 years of age died during hospitalization, 12% of persons 5–59 years of age and 38% of persons ≥60 years of age died while hospitalized (p<0.001).

A total of 16,582 of SARI case-patients were tested for influenza. Of these, 8,158 (49%) were female. More persons were tested during the 2009–2010 pandemic (9,879) than during 2011–2012 (6,703). Of the 16,597 case-patients tested, 3,352 (20%) tested positive for influenza (Table).

**Table:** Influenza-associated severe acute respiratory infections hospitalizations and deaths in Costa Rica, 2009–2012

Year and age group, y	Population size	No. hospitalizations for SARI†	No. deaths‡	No. (%) influenza-positive respiratory samples§	Influenza-associated hospitalizations			Influenza-associated deaths	
					No. (95% CI)¶	Rate# (95% CI)¶		No. (95% CI)¶	Rate** (95% CI)¶
2009									
<5	356,266	2,196	17	194/1,349 (14)	436 (336–535)	1.2 (0.9–1.5)		2 (1–3)	0.6 (0.3–0.8)
5–59	3,700,000	2,998	328	1,608/3,653 (44)	1,852 (1,737–1,977)	0.5 (0.47–0.53)		115 (95–138)	3.1 (2.6–3.7)
≥60	415,210	2,797	1,042	265/1,306 (20)	657 (513–804)	1.6 (1.2–1.9)		171 (112–231)	41 (27–56)
2010									
<5	350,902	2,583	27	185/1,230 (15)	529 (366–688)	1.5 (1.0–2.0)		4 (2–4)	1.1 (0.6–1.1)
5–59	3,800,000	2,595	317	507/1,472 (34)	1,185 (993–1,378)	0.3 (0.3–0.4)		107 (78–133)	2.8 (2.1–3.5)
≥60	444,981	3,073	1,172	161/879 (18)	742 (467–1,019)	1.7 (1.0–2.3)		229 (123–340)	51 (28–76)
2011									
<5	347,888	2,204	32	48/1,797 (3)	114 (67–161)	0.3 (0.2–0.5)		0	0
5–59	3,800,000	2,224	330	40/700 (6)	174 (72–286)	0.05 (0.02–0.08)		20 (6–38)	0.5 (0.2–1.0)
≥60	463,100	3,049	1,183	27/639 (4)	163 (53–279)	0.4 (0.1–0.6)		52 (12–96)	11 (3–21)
2012									
<5	344,577	1,927	22	79/1,526 (5)	164 (109–216)	0.5 (0.3–0.6)		1 (0–2)	0.3 (0.0–0.6)
5–59	3,900,000	2,016	229	162/1,034 (16)	423 (307–540)	0.1 (0.08–0.14)		31 (18–46)	0.8 (0.5–1.2)
≥60	481,557	2,695	1,051	76/1,012 (8)	260 (145–381)	0.5 (0.3–0.8)		71 (27–118)	15 (6–25)
Total	18,404,481	30,357	5,750	3,352/16,597 (20)	6,699 (5,165–8,264)	0.4 (0.3–0.4)		803 (474–1,149)	4 (3–6)

*SARI, severe acute respiratory illness. Population estimates based on Costa Rica census projections. †Number of persons hospitalized during 2009–2012 for SARI proxy diagnoses (ICD-10 code J9–18) (*8*) in any of 6 discharge diagnostic fields. ‡In-hospital deaths among persons hospitalized during 2009–2012 for SARI (International Classification of Diseases, 10th Revision, code J9–18) in any of 6 discharge diagnostic fields. §Annual number of nasal and pharyngeal specimens positive for influenza by immunofluorescence and PCR over total number tested. ¶Estimated through the product of the proportion of samples testing positive for influenza and the number of persons hospitalized for or inpatient deaths from SARI each month and sum of the products by age group, Costarricense de Seguro Social, San José, Costa Rica. #Per 1,000 person-years. **Per 100,000 person-years.

We estimated that 6,699 (95% CI 5,165–8,264) influenza hospitalizations occurred among all age groups (Table). These represented 0.4 hospitalizations for influenza per 1,000 person-years (py) (95% CI 0.3–0.4/1,000py). The rate of influenza-associated hospitalizations was higher during the 2009–2010 pandemic (mean 0.6/1000 py) compared with those during 2011– 2012 (mean 0.1/1000 py) (p<0.001) (Figure).

We estimated that 803 (95% CI 474–1,149) in-hospital deaths attributed to influenza occurred among all age groups (Table); 628 (78%) occurred during the 2009-2010 pandemic years. Influenza-associated deaths represented 12% of influenza-associated SARI hospitalizations and a rate of 4/100,000 py. Deaths occurred primarily among persons ≥60 years of age, for whom the influenza mortality rate was 29/100,000 py compared with 2/100,000 py among other age groups (p<0.001). During the 2009–2010 pandemic, 47 persons ≥60 years of age per 100,000 py died as a result of influenza-associated SARI, compared with 3 persons <60 years of age per 100,000 py.

## Conclusions

Our study suggests that Costa Rica had substantive numbers of influenza-associated hospitalizations and deaths. Hospitalization rates were highest among children <5 years of age and persons ≥60 of age. Although deaths increased during the pandemic among persons 5–59 years of age, persons ≥60 years of age were more likely to die as a result of influenza. Our findings support the decision of the officials of Costa Rican Social Security Board to provide seasonal influenza vaccination for children and older adults, and for groups that may be different during pandemics.

The influenza-associated hospitalization and mortality rates in our study were within the range of those estimated in middle-income countries in the American tropics. Pandemic influenza-associated hospitalization rates among children in Costa Rica were within the range of the influenza-associated severe pneumonia hospitalization rates in El Salvador among children <5 years of age (*9*), but higher than rates estimated from population-based surveillance in Guatemala (0.3/1000 py) (*11*). The rates calculated for this study were lower than those estimated for deaths from pneumonia associated with influenza (1.4, 95% CI 0.7–2.1 /100,000 py) (*12*). Our estimated influenza-associated mortality rates were similar, however, to deaths attributed to influenza-associated respiratory system failure in Mexico (3.7, [95% CI 3.0–4.4/100,000 population) (*13*). Rates were generated by using different case definitions, surveillance, and analytical methods. Standardization of surveillance (*6*) and the methods to estimate burden of disease may yield estimates more readily compared among countries (*8*).

This study had several limitations. We were unable to directly measure rates of laboratory-confirmed influenza among all hospitalized case-patients. Respiratory samples were from clinical specimens gathered during routine practice and from systematically identified patients. A different proportion of persons discharged from the hospital or who died after receiving a diagnosis of SARI may have tested positive for influenza than the proportion tested by convenience. Persons with respiratory illness may have been more likely to be admitted during the pandemic, thus inflating hospitalization rates during 2009–2010 when compared with 2011–2012. Also, a larger but unquantified proportion of samples were first tested through indirect immunofluorescence rather than the more sensitive PCR during 2011–2012 than during the pandemic (*14*).

Our study suggests that Costa Rica had substantive influenza-associated hospitalization and mortality rates, particularly among the very young and the elderly. Influenza-associated hospitalization and mortality rates were similar to those of neighboring countries that routinely dispense influenza vaccines and oseltamivir. Further studies may be warranted to explore the value and sustainability of expanded influenza vaccination programs among populations most at risk for development of severe illness**.**

Technical Appendix National Influenza Centre and influenza sentinel surveillance sites in the provinces of Costa Rica.

## References

[1] Cheng PY, Palekar R, Azziz-Baumgartner E, Marinho F, Iuliano D, Glew P, Regional estimates of influenza mortality. Achievements and future challenges in the surveillance of respiratory viruses; 2013 January 29–30, 2013; San Jose (Costa Rica): US Centers for Disease Control and Prevention; 2013.

[2] Centers for Disease Control and Prevention. CDC Says “Take 3” Actions to fight the flu. 2010 [updated 2014 Feb 12; cited 2013 Nov 1]. http://www.cdc.gov/flu/protect/preventing.htm

[3] World_Bank. How we Classify Countries. 2010 [cited 2010 Dec 5]. http://data.worldbank.org/about/country-classifications

[4] Ropero-Álvarez A, Kurtis HJ, Danovaro-Holliday M, Ruiz-Matus C, Andrus J. Expansion of seasonal influenza vaccination in the Americas. BMC Public Health. 2009;9:361–9. 10.1186/1471-2458-9-361 19778430 PMC2764707

[5] World Health Organization. International Classification of Diseases (ICD) 2011 [cited 2012 Sep 2]. http://www.who.int/classifications/icd/en/

[6] Pan American Health Organization-Centers for Disease Control and Prevention. Generic Protocol for Influenza Surveillance 2006 [cited 2012 Jul 15]. http://www1.paho.org/English/AD/DPC/CD/flu-snl-gpis.pdf

[7] Instituto Nacional de Estadística y Censo CR. Poblaciones. 2000 [cited 2013 Nov 1]. http://www.inec.go.cr/Web/Home/GeneradorPagina.aspx

[8] Nair H, Campbell H, Mounts A. A manual for estimating disease burden associated with seasonal influenza in a population. Geneva: World Health Organization; 2009.

[9] Clara W, Armero J, Rodriguez D, Lozano C, Bonilla L, Minaya P, Estimated incidence of influenza-associated severe pneumonia rates in children younger than 5 years in El Salvador, 2008–2010. Bull World Health Organ. 2012;90:756–63 . 10.2471/BLT.11.098202 23109743 PMC3471049

[10] Writing Committee of the WHO. Clinical aspects of pandemic (H1N1) 2009 influenza. N Engl J Med. 2010;362:1708–19. 10.1056/NEJMra1000449 20445182

[11] Reyes L, Arvelo W, Estevez A, Gray J, Moir J, Gordillo B, Population-based surveillance for 2009 pandemic influenza A (H1N1) virus in Guatemala, 2009. Influenza Other Respi Viruses. 2010;4:129–40. 10.1111/j.1750-2659.2010.00138.x 20409209 PMC4986580

[12] Freitas FT, Souza LR, Azziz-Baumgartner E, Cheng PY, Zhou H, Widdowson MA, Influenza-associated excess mortality in southern Brazil, 1980–2008. Epidemiol Infect. 2013;141:1731–40. 10.1017/S0950268812002221 23040669 PMC9151596

[13] Charu V, Chowell G, Mejia L, Echevarria-Zuno S, Borja-Aburto V, Simonsen L, Mortality burden of the A/H1N1 pandemic in Mexico: a comparison of deaths and years of life lost to seasonal influenza. Clin Infect Dis. 2011;53:985–93. 10.1093/cid/cir644 21976464 PMC3202315

[14] Atmar R, Baxter B, Dominguez E, Taber L. Comparison of reverse transcription-PCR with tissue culture and other rapid diagnostic assays for detection of type A influenza virus. J Clin Microbiol. 1996;34:2604–6. 8880531 10.1128/jcm.34.10.2604-2606.1996 PMC229331

