# Supplementary material for: Influenza-associated Hospitalizations and Deaths, Costa Rica, 2009–2012
Source: Emerg Infect Dis. 2014 May;20(5):878–81. doi: 10.3201/eid2005.131775 (PMC4012819; doi:10.3201/eid2005.131775)
Supplement: Technical Appendix — National Influenza Centre and influenza sentinel surveillance sites in the provinces of Costa Rica. [file 13-1775-Techapp-s1.pdf]

Article DOI: http://dx.doi.org/10.3201/eid2005.131775

Influenza-associated Hospitalizations and Deaths in Costa Rica, 2009–2012

Technical Appendix

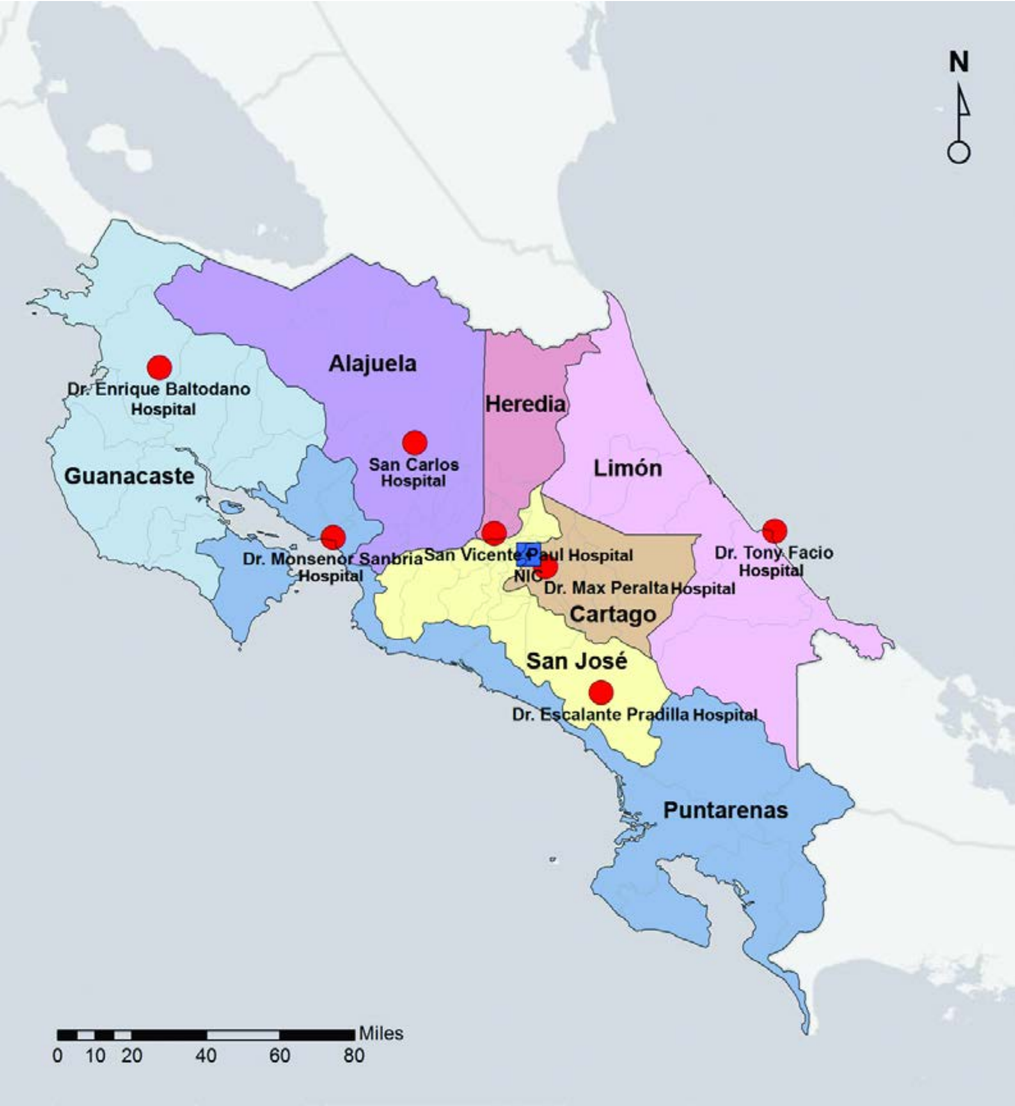

Technical Appendix Figure. National Influenza Centre and influenza sentinel surveillance sites in the provinces of Costa Rica. Square indicates National Influenza Center; circles indicate sentinel surveillance sites. Map produced by the Pan American Health Organization, Communicable Disease and Health Analysis, International Regulation by using Esri software (http://www.esri.com).
